# Treatment of Glioma Using neuroArm Surgical System

**DOI:** 10.1155/2016/9734512

**Published:** 2016-05-24

**Authors:** Yaser Maddahi, Kourosh Zareinia, Liu Shi Gan, Christina Sutherland, Sanju Lama, Garnette R. Sutherland

**Affiliations:** Project neuroArm, Department of Clinical Neuroscience and the Hotchkiss Brain Institute, University of Calgary, 3280 Hospital Drive NW, Calgary, AB, Canada T2N 4Z6

## Abstract

The use of robotic technology in the surgical treatment of brain tumour promises increased precision and accuracy in the performance of surgery. Robotic manipulators may allow superior access to narrow surgical corridors compared to freehand or conventional neurosurgery. This paper reports values and ranges of tool-tissue interaction forces during the performance of glioma surgery using an MR compatible, image-guided neurosurgical robot called neuroArm. The system, capable of microsurgery and stereotaxy, was used in the surgical resection of glioma in seven cases. neuroArm is equipped with force sensors at the end-effector allowing quantification of tool-tissue interaction forces and transmits force of dissection to the surgeon sited at a remote workstation that includes a haptic interface. Interaction forces between the tool tips and the brain tissue were measured for each procedure, and the peak forces were quantified. Results showed maximum and minimum peak force values of 2.89 N (anaplastic astrocytoma, WHO grade III) and 0.50 N (anaplastic oligodendroglioma, WHO grade III), respectively, with the mean of peak forces varying from case to case, depending on type of the glioma. Mean values of the peak forces varied in range of 1.27 N (anaplastic astrocytoma, WHO grade III) to 1.89 N (glioblastoma with oligodendroglial component, WHO grade IV). In some cases, ANOVA test failed to reject the null hypothesis of equality in means of the peak forces measured. However, we could not find a relationship between forces exerted to the pathological tissue and its size, type, or location.

## 1. Introduction

The complexity of the central nervous system and neural network makes surgical intervention for neurosurgical disease somewhat complex [1, 2]. Surgical treatment glioma remains a significant challenge due to their infiltrative nature and relationship with the normal brain [3]. Therefore, surgeons face a dilemma relative to decisions on extent of resection in glioma surgery versus the risk of neurological deficit and hence quality of life. As a result for the majority of patients with glioma, surgical resection is incomplete and recurrence inevitable [4–8]. In addition, dissecting the tumour/brain interface may result in movement of glioma cells into the adjacent normal brain [9]. Hence, using instrumented surgical tools to enhance tumour resection is fundamental to achieve optimal outcome in glioma surgery. Intraoperative magnetic resonance imaging (iMRI) systems have been translated into the neurosurgical operating room (OR) for lesion localization and resection control [10, 11]. More recently, image-guided robotic technologies have been translated into neurosurgical procedures for increased precision and accuracy [12–14]. Robotic technology offers several advantages over conventional surgery including (i) combining decision-making capability of the human brain with the precision and accuracy of machine technology, (ii) facilitating surgery at smaller scale by providing access to narrower surgical corridors, (iii) eliminating the problem of brain shifting by combining surgery with imaging, either updated or in real time, and (iv) enhancing surgical performance through the provision of motion and force scaling, together with virtual fixtures.

A number of robotic systems with different technical specifications have been developed [15–19]. There exist five prototyped systems for microsurgery, none of which is commercially available, and only three of them can provide force feedback or haptic sensation (NeuRobot [18], ROBOCAST [17], and neuroArm [12]). Two systems have been used for conducting operations on human (neuroArm and NeuRobot), and the only system which remains in clinical use is neuroArm, with 69 successful operations. This report provides information of tool-tissue interaction forces during performance of glioma procedures using neuroArm. These forces were measured and analyzed during performance of seven glioma operations. Peak forces during each neurosurgical task were calculated, and a set of comparative studies between the seven cases was performed. Statistical analysis was also conducted to test if there is a correlation between the size, type, or location of the pathological tissue and the forces measured. This paper extends the work previously performed by the authors to quantify the forces during performance of various neurosurgical operations such as grade III oligoastrocytoma and meningioma [19] and a single case of glioma [20]. The information of quantified forces may be used as a reference for training surgeons and neurosurgery residents in the performance of surgery using robotics with haptic interface.

This paper is organized as follows.  Section 2 presents the experimental setup, followed by explaining the coordinate systems attached to the robotic manipulator. Neurosurgical procedures and corresponding surgical tasks are described in Section 3. Experimental results are presented in Section 4. Discussions and conclusions are outlined in Sections 5 and 6.

## 2. Experimental Setup

**Figure 1:**
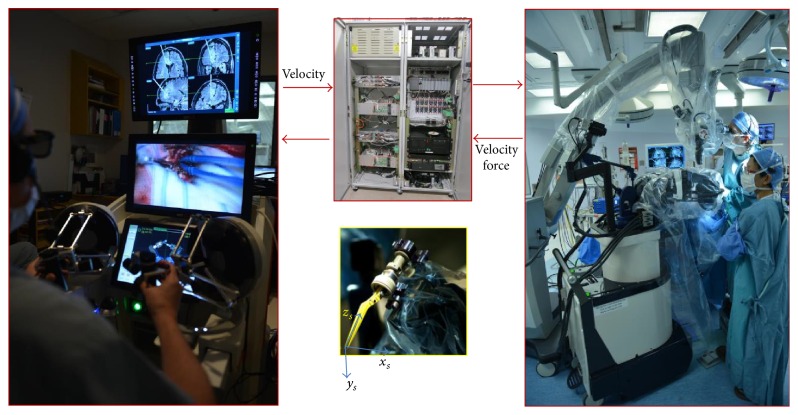
The surgical setup inside OR (right) including neuroArm robotic manipulators located above the patient's head, a surgical microscope, and an assistant surgeon. The surgeon uses two Omega. 7 haptic hand-controllers (left) to command the manipulators to move inside the surgical zone. The inset in the middle shows coordinate system attached to the end-effector of the right manipulator (bottom) and the controller cabinet (top).

The experimental setup consists of two MR compatible manipulators, capable of stereotaxy and microsurgery (see Figure 1). The manipulators are made of titanium, polyoxymethylene, and polyetheretherketone and are actuated by ultrasonic piezoelectric motors [21, 22]. They are mounted on a height-adjustable mobile base, which includes a field camera and digitizing arm. A set of neurosurgical tools are specifically designed to be MR-safe and attachable to the tool holder. The main system control cabinet includes delta-tau controllers, motor drivers, power supplies, a heart-beat monitor, and a PLC. As shown in Figure 1, the workstation consists of two high definition medical grade monitors (LMD 2450 MD, Sony, Japan), one for display of MR images and second for stereoscopic view of the surgical site linked to two HD cameras mounted on the surgical microscope. The workstation comprises a robot control user interface on a 15-inch touchscreen for virtual display of the robotic manipulators and relay of commands and monitors the status of the robot. Moreover, the workstation is equipped with a set of foot pedals (clutches) to send/stop sending commands to the robotic arms.

A surgeon sits at the workstation and utilizes two haptic hand-controllers to send motion information to the slave manipulators. Velocity of the hand-controllers over a time interval, in the digitized system, is translated into the displacement and is then mapped into the coordinate system attached to each end-effector. The velocities are then converted into motor signals using Jacobian of the robotic arms. The actual displacement of the arms, measured by absolute encoders, is sent to the master site to update real-time position and orientation of arms and augmented virtual reality arms. Tool-tip interaction forces are measured by two Nano17 force sensors mounted on each end-effector and in contact with the tool. The force signals are sent to the master side to be regenerated by the haptic hand-controllers. The network channel is wired Ethernet; therefore, there are no lag, packet loss, and packet order during communication [23]. The haptic hand-controllers on the workstation are capable of providing 12 N of continuous force, which covers the range required in neurosurgery [19]. This setup provides an adequate telepresence for the surgeon in terms of touch and haptic feedback.

**Figure 2:**
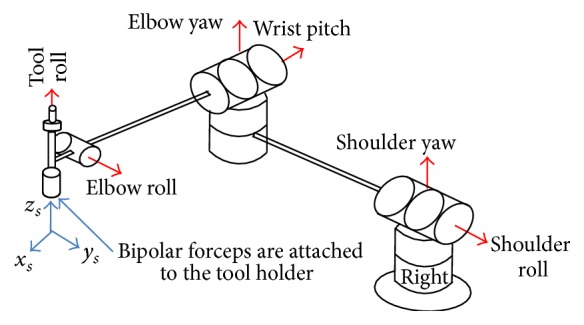
Diagram of the right arm of the neuroArm surgical system.

Figure 2 illustrates the coordinate systems attached to the joints and the end-effector of the right arm of the neuroArm surgical system. As observed, each robotic arm has six revolute joints to provide a complete range of motion. Motion of the surgical tool (bipolar forceps) is defined by three translational components along the {*x*_*s*_ *y*_*s*_ *z*_*s*_} frame and three rotational components about this frame [19, 24]. The mapping system and corresponding matrices are explained by the authors in [25]. The neuroArm system is able to hold two different surgical tools, each by one of the manipulators. For this study, the tools utilized are a suction tool on the left manipulator and bipolar forceps on the right manipulator. As the bipolar forceps are used as the primary surgical tool for manipulation/dissection of brain tissue and the suction tool is only used for suctioning excessive fluids such as blood and water from surgical site, only results of the forces measured at the forceps tips are reported.

## 3. Test Procedure

This report presents the use of neuroArm surgical system as an adjunct to neurosurgery in the resection of glioma in seven cases. Descriptive information for each patient is reported in Table 1. An experienced senior surgeon (GS) as the primary surgeon together with an assistant surgeon (neurosurgical resident) performed all seven procedures. Since the length of use of the robotic system differed across the seven cases, 1000-second force data were extracted from each case for analysis, totaling about 2 hours of force data for all 7 cases. Each patient provided informed consent, and all surgical procedures were performed at the Foothills Medical Center, Calgary, Alberta, Canada. All procedures were performed in compliance with the University of Calgary Conjoint Health Research Ethics Board of the Faculties of Medicine, Nursing, and Kinesiology and with Health Canada.

**Figure 3:**
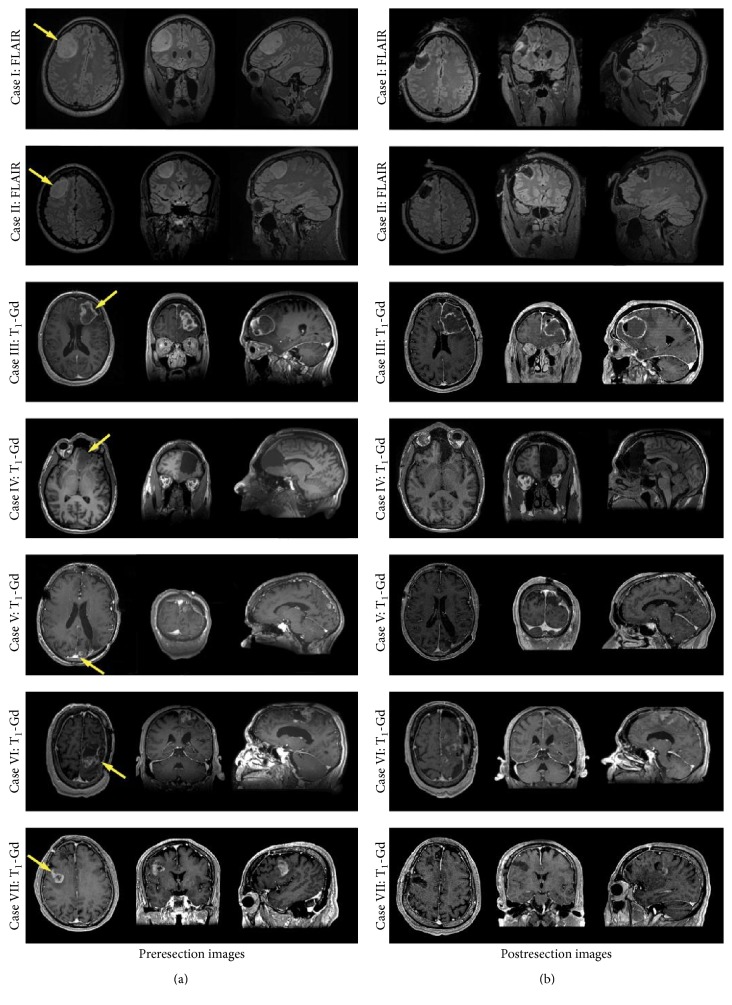
((a) and (b)) Pre- and postresection MR images (representative axial, coronal, and sagittal views); different MR imaging sequences have been included to highlight the various tumours. Three of the patients (Cases I, II, and IV) had nonenhancing glioma WHO grade III and four patients (Cases III and V–VII) enhancing lesions following administration of gadolinium contrast agent. Postresection MR images show the extent of resection for each patient. Some residual enhancement is evident in patients I, VI, and VII.

The pre- and postoperative MR images of each patient are shown in Figure 3. The tumours varied in location and size across the seven patients (Figure 3 and Table 1). For each case, positioning of the neuroArm robot was important as it is patient-specific, varying with the side and location of the tumour. Considerable preoperative planning was also required to ensure that the positions of the robotic manipulators and operating microscope are well aligned with the assistant surgeon's operative field and space, the scrub nurse's workspace, and general OR workflow. The primary surgeon at the workstation, the assistant surgeon, scrub nurse, and the rotating OR nurse each wore a pair of headsets with microphones for communication. In all the cases, following sterile draping of the robot, bipolar forceps were attached to the right manipulator and suction tool was attached to the left.

The tasks conducted in each procedure consisted of (i) manipulation: pushing motion for assessing tissue consistency, lateral retraction, gentle spreading apart of tissue, and structure manipulation; (ii) coagulating: tumour-brain interface, tumour, and vessels; and (iii) placement: placement of cotton strips for hemostasis and protecting structures. The tasks were not performed in a particular order, and each task was repeated multiple times in a given procedure.

## 4. Results

Peak values of each force signal were used to compute the mean and the standard deviation (SD) values. Results of measuring the forces for four robot-assisted neurosurgical cases, studied by the authors, have shown that the forces exerted by the suction tool, in neuroArm manipulators, are less than those measured for the bipolar forceps, which was expected given that the surgeon is right-handed [19]. In addition, in neurosurgical procedures, the bipolar forceps are used as the primary tool, while the suction is employed to remove fluids and tissue debris and sometime for gentle retraction. In this paper, only the forces of the bipolar forceps are reported, as we aim to quantify the peak forces along with the maximum forces applied during these seven clinical studies.

**Figure 4:**
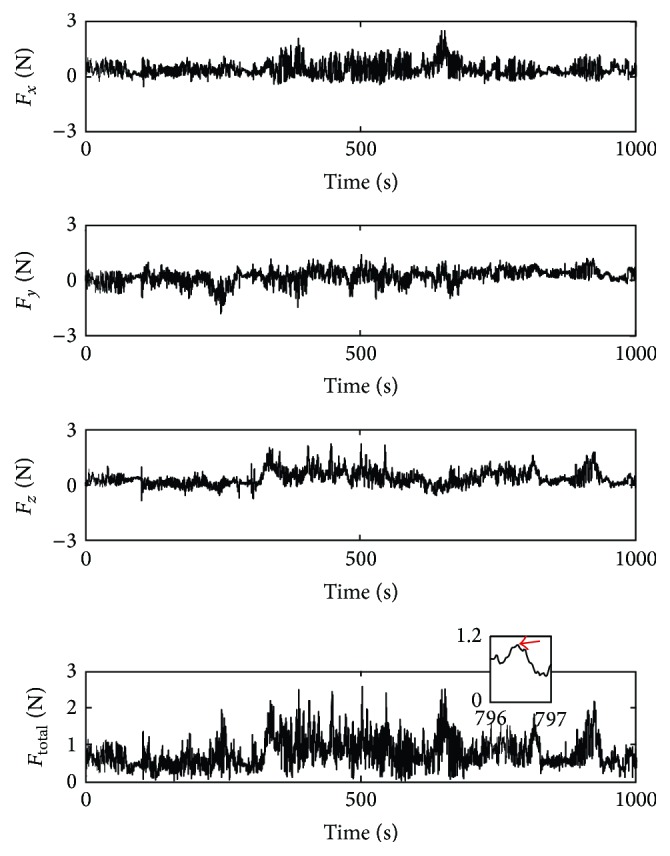
Forces measured during performance of Case I over 1000 seconds of robot-assisted neurosurgery. The inset shows how the peak force is determined for a time interval of 1 second.

Figure 4 shows interaction force components between the bipolar forceps tips and the brain tissue during the performance of Case I (anaplastic oligoastrocytoma, WHO grade III). For this typical case, maximum value of the resultant forces was 2.57 N and the mean force (+SD) was 1.36 ± 0.47 N. The peak resultant force was calculated using the sum of squares of the force components in the *x*_*s*_, *y*_*s*_, and *z*_*s*_ directions, $F=\sqrt{F_{x}^{2}+F_{y}^{2}+F_{z}^{2}}$. The method of computing values of peak forces is also pictorially shown in the inset in Figure 4. Specifically, a MATLAB program was developed to detect peak values in each force signal. In the program, when the sign of the first derivative of the force signal changes from positive to negative, the local maxima value is considered as the peak force.

**Figure 5:**
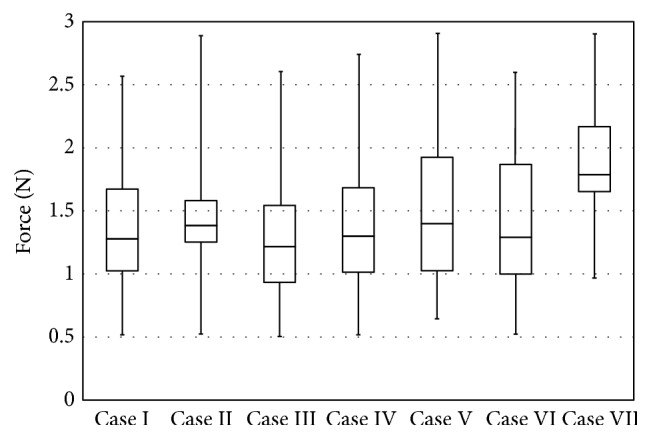
Box and whisker plot showing the distribution of peak forces over 1000-second period of robot-assisted surgery for each of the glioma cases.

Table 2 presents the mean values (±SD) of the absolute forces at the right manipulator end-effector which holds the bipolar forceps. The minimum and maximum peak forces across seven surgical cases were observed in Case III (0.50 N) and Case II (2.89 N), respectively. However, the mean peak forces of Cases I, II, III, IV, and VI were almost the same. In addition, the maximum peak forces of Cases II, V, and VII were very close. Although the maximal peak force was observed in Case II, the forces from this surgical procedure were the least variable. The mean forces of Case VII were much higher than other procedures; however, the value of SD was comparable with other cases. The peak forces in all procedures varied from 2.57 N (Case I) to 2.89 N (Case II) and the mean values changed from 1.27 N (Case III) to 1.89 N (Case VII). The number of peak forces (sampled data) considered for analysis is also shown in Table 2.  Figure 5 shows the full range (from minimum to maximum peak force) of variations in peak forces quantified for all seven cases. This figure enables us to study the distributional characteristics of peak forces measured during each robot-assisted surgery.

## 5. Discussion

**Figure 6:**
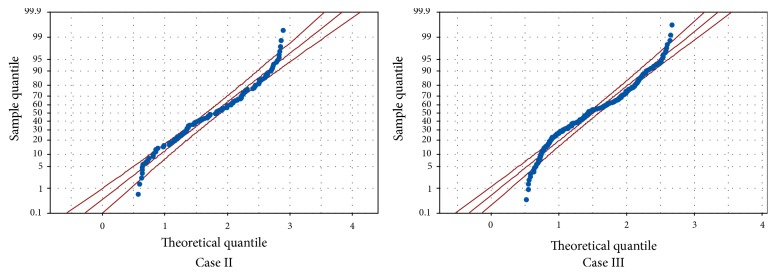
Q-Q plots of forces quantified during performance of procedures II and III. As observed, in both cases, the force data points follow a linear pattern.

Analysis of Variance (ANOVA) was used to test differences between peak forces measured in all seven surgical cases. We employed one-way ANOVA to test general rather than specific differences among mean values of forces. *p* values less than 0.05 (*p* < 0.05) were considered to be statistically significant [26]. In all seven procedures, the normality of force data was first confirmed by measuring the skewness and kurtosis indices as well as sketching the Q-Q plot. Skewness and kurtosis values of more than 2 and less than −2 do not satisfy the normality and indicate that the test used to measure the trait (quantified forces) is not appropriate for the proposed application [27]. The values of both skewness and kurtosis measures are reported in Table 2. As observed, the skewness and kurtosis values for all procedures confirm that the peak forces satisfy the normal distribution criterion. Quantile-quantile (Q-Q) plot of two typical force sets (Cases II and III) is shown in Figure 6. As observed, both force datasets follow almost a linear pattern in the center; however, some outliers are seen indicating deviation from the line at the tail of the distribution. Similar observations were detected for the forces measured in other procedures; that is, force data followed almost a linear pattern.  Table 3 lists the *p* values obtained from ANOVA test. As observed, in some pairs of surgical cases, there is insufficient evidence at the 5% level of significance to support that there is a difference in mean values of forces, such as Cases I and IV. It means that we could not detect different mean values in the populations obtained from each pair of force data. For example, the *p* value obtained by comparing the peak forces in Cases I and IV is 0.824 that is much more than 0.05 (see the bold font cell). However, in some pairs such as Cases I and II or Cases II and III, unequal mean values of forces were detected. As expected from Figure 5 and Table 2, Case VII had a significant difference in force means with all surgical procedures; that is, the *p* values were much less than 0.05.

In practice, surgeons normally find oligodendroglioma of relatively softer consistency and high grade (GBM/anaplastic astrocytoma) of slightly firmer consistency, although high grade glioma may have necrotic components, which are very soft and readily removed with suction. However, in Case VII (glioblastoma with oligodendroglial component WHO grade IV), a higher number of peak forces were observed. One reason to experience increased forces may relate to the tumour vascularity necessitating more aggressive and faster coagulation. Moreover, different robot position with respect to the patient's head, dissimilar tumour location and depth, and glioma subtype and grade could be affecting the amount of peak forces during each surgical procedure.

## 6. Conclusion

This paper reported the amount and range of interaction forces observed during performance of seven neurosurgical cases that were conducted to resect glioma. All operations were performed using the neuroArm robotic system. The tool-tissue interaction forces between the bipolar forceps tips and the brain tissue were quantified using two titanium Nano17 force sensors attached to each tool holder of the robot. Peak values of each force signal were calculated for further analysis. Maximum and minimum peak forces of 2.89 N and 0.50 N were observed, respectively. The ANOVA test was performed to determine whether the mean forces measured in all seven procedures were significantly different. Results failed to reject the hypothesis of equality of mean values in two populations of peak forces in some cases; however, forces in Case VII had a significant difference in means with other cases. Some reasons to observe different values of peak forces, mean values, and SD could be (i) different robot position, in each case, with respect to the patient's head, (ii) different tumour depth, location, and size, (iii) glioma subtype and grade, and (iv) tumour vascularity necessitating more aggressive and faster coagulation that may translate to increased force. Future work will focus on collecting the force information from a larger number of cases with different tumour subtypes, WHO grades, size, and location. Recorded force data may be of value for surgical education and case rehearsal and can contribute to the development of neurosurgical simulators whereby information of tool-tissue interaction forces will allow modeling of brain tissue in health and disease.

## Figures and Tables

**Table 1:** Patient-specific information.

Case	Year	Gender	Age	Type	Surgical zone	Tumour size (cm^3^)
I	2013	M	33	Anaplastic oligoastrocytoma, WHO grade III	Right frontal lobe	3.7 × 4.5 × 4.3
II	2013	M	30	Anaplastic astrocytoma, WHO grade III	Right frontal lobe	4.3 × 2.5 × 3.5
III	2013	F	60	Anaplastic oligoastrocytoma, WHO grade III	Right frontal lobe	4.8 × 3.8 × 4.5
IV	2014	M	36	Oligoastrocytoma, WHO grade II	Left frontal lobe	5.2 × 2.0 × 2.1
V	2014	F	64	Anaplastic oligodendroglioma, WHO grade III	Left parietal/occipital lobes	0.8 × 0.7 × 1.0
VI	2014	M	58	Anaplastic oligodendroglioma WHO grade III	Left frontal/parietal lobes	3.1 × 1.5 × 3.2
VII	2015	M	59	Glioblastoma with oligodendroglial component WHO grade IV	Right frontal lobe	3.1 × 2.0 × 3.3

**Table 2:** Statistical indices of peak forces of the bipolar forceps over 1000-second period of robot-assisted surgery for each glioma case.

	Case I	Case II	Case III	Case IV	Case V	Case VI	Case VII
Number of peak forces	127	123	201	153	177	135	117
Max	2.57	2.89	2.68	2.75	2.88	2.68	2.87
Min	0.52	0.52	0.50	0.52	0.64	0.52	0.97
Mean ± SD	1.36 ± 0.47	1.50 ± 0.40	1.27 ± 0.44	1.37 ± 0.49	1.52 ± 0.58	1.41 ± 0.56	1.89 ± 0.41
Skewness	0.26	−1.68	−0.60	0.81	1.65	−1.32	−1.95
Kurtosis	1.20	−1.42	0.74	0.09	0.97	1.01	1.68

**Table 3:** The *p* values obtained using ANOVA for each pair of surgical forces.

	Case II (*n* = 123)	Case III (*n* = 201)	Case IV (*n* = 153)	Case V (*n* = 177)	Case VI (*n* = 135)	Case VII (*n* = 117)
Case I (*n* = 127)	0.016	0.069	**0.824**	0.013	0.466	<0.01^*∗*^
Case II		<0.01	0.028	0.741	0.151	<0.01
Case III			0.033	<0.01	0.011	<0.01
Case IV				0.017	0.585	<0.01
Case V					0.095	<0.01
Case VI						<0.01

^*∗*^Small numbers of *p* values are shown as <0.01.
